# Enantioselective Human Serum Albumin Binding of Apremilast: Liquid Chromatographic, Fluorescence and Molecular Docking Study

**DOI:** 10.3390/ijms24032168

**Published:** 2023-01-21

**Authors:** Gergely Dombi, Péter Horváth, Béla Fiser, Arash Mirzahosseini, Máté Dobó, Zoltán-István Szabó, Gergő Tóth

**Affiliations:** 1 Department of Pharmaceutical Chemistry, Semmelweis University, H-1085 Budapest, Hungary; 2 Higher Education and Industrial Cooperation Centre, University of Miskolc, Egyetemváros, H-3515 Miskolc, Hungary; 3 Department of Biology and Chemistry, Ferenc Rakoczi II Transcarpathian Hungarian College of Higher Education, Transcarpathia, 90200 Beregszasz, Ukraine; 4 Department of Physical Chemistry, Faculty of Chemistry, University of Lodz, 90-149 Łódź, Poland; 5 Department of Pharmaceutical Industry and Management, Faculty of Pharmacy, George Emil Palade University of Medicine, Pharmacy, Science, and Technology of Targu Mures, 540142 Targu Mures, Romania; 6 Sz-imfidum Ltd., 525401 Lunga, Romania

**Keywords:** apremilast, drug delivery, HSA binding, chiral chromatography

## Abstract

The interaction between human serum albumin (HSA) and apremilast (APR), a novel antipsoriatic drug, was characterized by multimodal analytical techniques including high-performance liquid chromatography (HPLC), fluorescence spectroscopy and molecular docking for the first time. Using an HSA chiral stationary phase, the APR enantiomers were well separated, indicating enantioselective binding between the protein and the analytes. The influence of chromatographic parameters—type and concentration of the organic modifier, buffer type, pH, ionic strength of the mobile phase, flow rate and column temperature—on the chromatographic responses (retention factor and selectivity) was analyzed in detail. The results revealed that the eutomer *S*-APR bound to the protein to a greater extent than the antipode. The classical van ’t Hoff method was applied for thermodynamic analysis, which indicated that the enantioseparation was enthalpy-controlled. The stability constants of the protein–enantiomer complexes, determined by fluorescence spectroscopy, were in accordance with the elution order observed in HPLC (*K_R_*_-APR-HSA_ = 6.45 × 10^3^ M^−1^, *K_S_*_-APR-HSA_ = 1.04 × 10^4^ M^−1^), showing that, indeed, the later-eluting *S*-APR displayed a stronger binding with HSA. Molecular docking was applied to study and analyze the interactions between HSA and the APR enantiomers at the atomic level. It was revealed that the most favored APR binding occurred at the border between domains I and II of HSA, and secondary interactions were responsible for the different binding strengths of the enantiomers.

## 1. Introduction

The majority of the currently marketed drugs contain at least one chiral center. Although some of them are sold as racemates, the pharmacodynamic and pharmacokinetic parameters of the enantiomers are inevitably different because of the intrinsic chiral nature of the human body. The classical example of differences in drug action is the notorious case of thalidomide; while *R*-thalidomide is a sedative, *S*-thalidomide is a teratogen [1,2,3]. Since proteins are also chiral in nature in terms of both their primary structure and their secondary or higher-order structures, they have the potential to discriminate between enantiomers [4,5,6]. Various types of proteins have been immobilized and used to prepare chiral stationary phases (CSPs) for liquid chromatography, ranging from enzymes to serum transport proteins and glycoproteins. Among these, the most important in practice are transport proteins such as human serum albumin (HSA), bovine serum albumin (BSA) and alpha1-acid glycoprotein (AGP) [4,7]. Apart from these three protein-based CSPs, there are only two other official chromatographic phases described in the US Pharmacopeia, based on ovomucoid and cellobiohydrolase. The role of protein-type columns in chiral separation has decreased in recent times. However, an advantage of protein-based columns is that the chromatographic results can also reflect the in vivo protein binding affinity of drugs [8]. Since at least 90% of drugs bind to plasma proteins, a high-throughput in vivo transport protein binding result is particularly important, as HSA binding highly affects the pharmacokinetics and bioavailability of a drug [9,10]. HSA is a globular protein, and its heart-shaped structure is composed of three main domains (I–III), each containing two subdomains (A and B), characterized by the presence of *α*-helices in the structure, with multiple ligand-binding sites localized in each of these subdomains [11,12]. According to Sudlow’s classification, there are two main binding sites for drug ligands of HSA, namely, Sudlow’s site I, positioned in subdomain IIA, and site II, positioned in subdomain IIIA. Site I is concerned with the selective binding of heterocyclic anions, such as ibuprofen, while aromatic carboxylates bind to site II. However, it should be noted that the binding of all the ligands cannot be described according to the Sudlow’s model. Site III, a third binding pocket located in subdomain IB, is a hydrophobic D-shaped cavity and is also used for interaction by some compounds such as bilirubin. Some studies suggested Sudlow’s site III as the third major site having the potential to bind drug ligands of HSA [13,14]. However, it should be noted that in vitro and in vivo binding data could be different, as, for example, the fatty acid content in plasma highly influences the binding properties. There are many methods to characterize protein–drug interactions: chromatographic techniques [15,16], circular dichroism [17,18], fluorescence [11,19] and nuclear magnetic resonance spectroscopy [20], as well as ultrafiltration [21], ultracentrifugation [22], calorimetric [23], UV-pH titration [24], etc. In addition, the characterization of stereoselective drug–protein interactions by computational methods may also be valuable to provide insights into chiral recognition mechanisms and to further design and optimize chiral separations [25]. Understanding the analyte–protein binding interactions on an atomic level can also aid further drug design [26].

**Figure 1:**
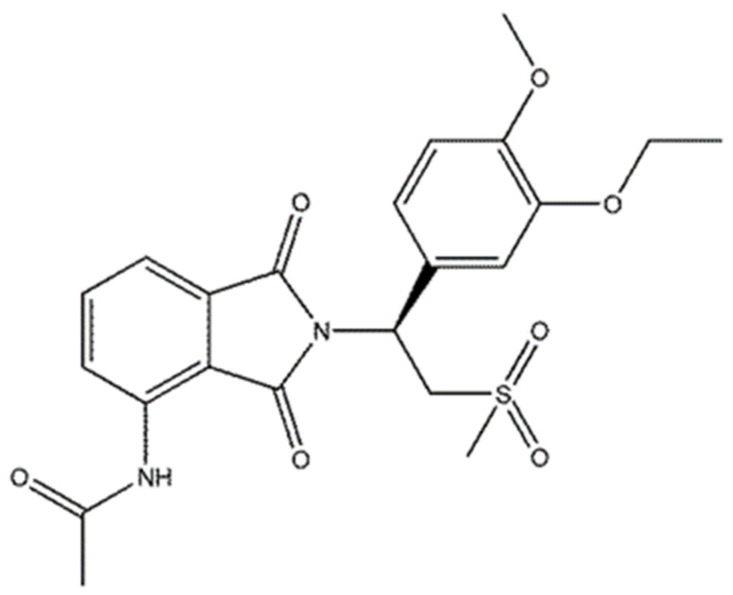
Structure of apremilast.

APR is a phthalimide analogue (Figure 1) that is used against psoriasis and psoriatic arthritis. It acts as a selective inhibitor of the enzyme phosphodiesterase-4 (PDE-4) and inhibits the spontaneous production of TNF-alpha from human rheumatoid synovial cells.

APR has one chiral center, and the more potent *S*-enantiomer is marketed. Based on the public EMEA assessment report of Otezla^®^, the overall mean APR percent bound was 88.6% in mouse plasma, 90.6% in rat plasma, 80.9% in rabbit plasma, 84.3% in monkey plasma and only 68.3% in human plasma in the tested concentration range from 0.25 to 2.5 μg/mL [27]. Plasma protein binding was much lower in humans compared to animals. In the literature, there are no data for albumin binding by APR, even though it would be of great utility for detecting possible drug–drug interactions. The present work aimed to scrutinize the stereoselective HSA binding by APR through HPLC, fluorescence spectroscopy and molecular docking. Using multimodal analytical techniques is necessary to eliminate the limitations of each method. Although HPLC separation on an HSA-bonded CSP has long been described for the analysis and the characterization of HSA binding by drugs, there are only a few articles in the literature that systematically investigate the correlation between mobile phase constituents and binding data. Therefore, another aim of this study was to determine the influence of different chromatographic parameters on the separation performance, as well as on protein binding. The APR-HSA interactions reported here explain the binding mechanism at the molecular level and will facilitate efforts to modify new therapeutic drugs and optimize their distribution within the human body.

## 2. Results and Discussion

### 2.1. HPLC Study

A Chiral-HSA column was employed to systematically study the enantiospecific HSA binding of APR and investigate the effects of several chromatographic conditions upon the separation performance.

#### 2.1.1. Buffer Type and Organic Modifier

During the initial experimental runs, we evaluated the impact of different buffer types on the enantioseparation performance. In the early studies described in the literature, acetate and phosphate buffers were used. The advantage of ammonium–acetate buffers is their compatibility with mass spectrometry [28,29,30], while several studies described that phosphate buffers mimic the physiological conditions of human plasma and, consequently, are being used in protein-binding studies [16,30]. The experiments were run at 25 °C, both buffers were set at pH = 7, and IPA was used as an organic modifier. The use of the phosphate buffer resulted in a better peak shape and higher resolution values than that of the ammonium acetate buffer; therefore, phosphate buffer was used throughout the study.

In the next step, different organic modifiers in different percentages were tested. Columns with immobilized proteins, such as Chiral-HSA, are very susceptible to the type and percentage of the organic modifier, which can lead to changes in the tridimensional structure of the protein and can alter the secondary interactions between the analyte and the CSP [30,31]. According to the instructions of the column manufacturer, the maximum usable organic modifier percentage in the mobile phase was 20% [32]. In our study, the effect of 2-propanol (IPA), 1-propanol (PROP), ethanol (EtOH), methanol (MeOH) and acetonitrile (ACN) on the retention factor (*k*) and selectivity (*α*) was investigated. The organic modifiers were used in five different percentages in 10 mM phosphate buffer adjusted to pH 7, using a flow rate of 0.7 mL/min, at 25 °C.

**Figure 2:**
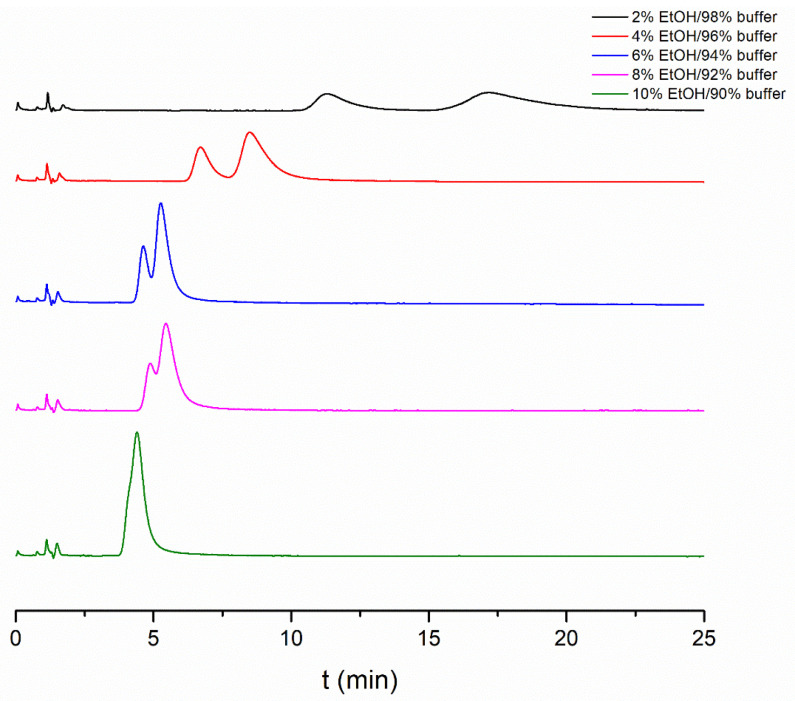
Effect of EtOH content in the mobile phase on enantioseparation of APR enantiomers. Chromatographic conditions: Chiral-HSA column, thermostated at 25 °C, flow rate 0.7 mL/min.

Enantioseparation was observed regardless of the applied modifier, and the enantiomer elution order was the same in all cases, *R* < *S*, meaning that the eutomer *S*-APR had a higher affinity with the protein. The obtained chromatographic data using different organic modifiers are presented in Table 1, while some representative chromatograms in different EtOH–buffer mixtures are depicted in Figure 2. As it can be observed, the highest retention factor was obtained in MeOH, while the lowest was in ACN. It can also be seen that there is an exponential relationship between the organic solvent content of the mobile phase and the retention factor. Thus, even a little amount of organic modifier could alter the protein binding. This also means that the type and proportion of the selected organic modifier should be considered when comparing measured data. Regarding enantioselectivity, the highest values were observed in MeOH and IPA. For choosing the most suitable organic modifier, analysis time, selectivity and column lifetime were all taken into consideration. Based on these aspects, 4 *v*/*v*% IPA was chosen, and further studies were conducted using a 96/4 *v*/*v*% phosphate buffer/IPA mixture.

#### 2.1.2. Buffer pH, Ionic Strength and Flow

**Figure 3:**
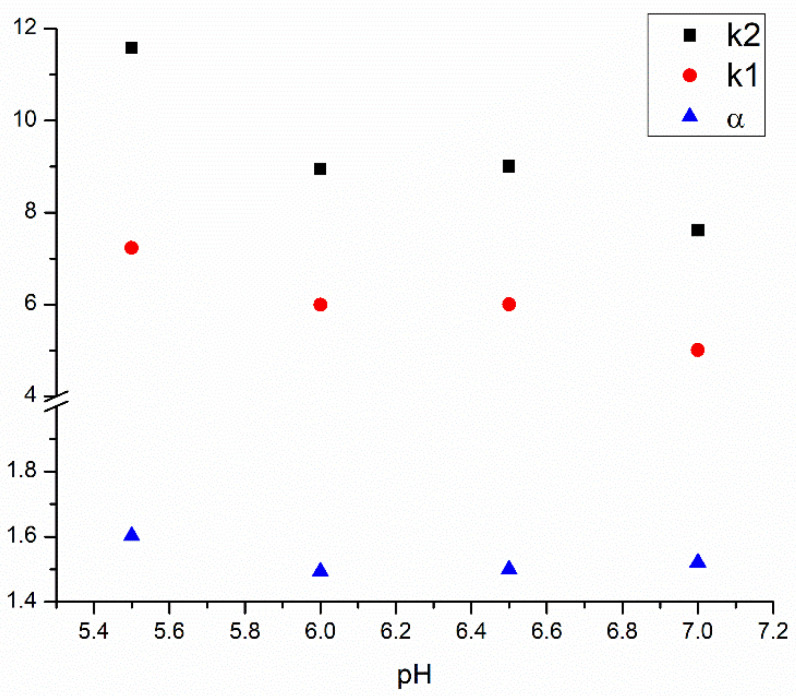
Influence of the pH of the phosphate buffer on the retention factors and enantioselectivity.

In the next steps, the effect of buffer pH and ionic strength upon separation performance was monitored. According to the manufacturer, the Chiral-HSA column is stable between pH 5 and 7 [32]. APR is a neutral molecule; therefore, its protonation state is independent of the pH. However, the protonation state of HSA differs at various pH values (its anionic character increases with pH), which can highly influence the binding process [16,33]. Four different pH values—5.5, 6.0, 6.5 and 7.0—were investigated, and in general, lower retention factors were obtained at higher pH values in IPA; however, due to a better peak shape, better resolution values were observed at lower pH (Figure 3). This experiment also underlines the necessity of an appropriate pH control in characterizing protein binding, regardless of the method.

As another step, the effect of the buffer’s ionic strength (10 mM, 20 mM, 30 mM, 40 mM phosphate with 4 *v*/*v*% IPA) was investigated. The relationship between ionic strength and retention and selectivity is not linear. Higher selectivity values were observed at 10 mM ionic strength in this range. The flow rate also varied between 0.5 and 0.8 mL/min. As expected, a higher flow rate caused lower retentions without a significant change in enantioselectivity.

Based on the obtained results, further experiments were performed with 10 mM phosphate buffer pH = 7/IPA 96/4 *v*/*v*%, with a 0.7 mL/min flow.

#### 2.1.3. Effect of the Temperature—Thermodynamic Analysis

The separation temperature is one of the main determinant factors that can affect selectivity, resolution, retention factor and even the elution order of enantiomers during HPLC enantioseparations [34,35]. To investigate the effects of temperature and to gain a better understanding of the mechanistic aspects of the enantiodiscrimination process on the Chiral-HSA column, thermodynamic analysis was performed in a 10 mM phosphate buffer/IPA 96/4 *v*/*v*% mixture.

To understand the energetic interactions between the analytes and the stationary phase, the classical van ’t Hoff approach was applied assuming only enantioselective interaction sites on the stationary phase. However, it should be noted that it is more realistic if both enantioselective and non-enantioselective interactions are considered [36,37].

Van ’t Hoff plots were constructed according to Equation (1) by plotting the natural logarithm of the retention factor as a function of the inverse of the absolute temperature in the temperature range of 10–30 °C, with 5 °C increments (a higher temperature was not applied due to possible column damage)

(1) $$\ln k=\frac{\Delta H^{{}^{\circ}}}{RT}+\frac{\Delta S^{{}^{\circ}}}{R}+\ln\Phi$$

where *R* stands for the universal gas constant, *T* is the temperature in Kelvin, and *k* is the retention factor of the individual enantiomers. Δ*H°* denotes the standard enthalpy, while Δ*S*° is the standard entropy change of transfer of the solute from the mobile phase to the stationary phase, and *Φ* is the phase ratio of the HSA column. If Δ*H°* is constant in the selected temperature range, a linear relationship is obtained between ln*k* and 1/T, with a slope of −Δ*H*°/*R* and an intercept of Δ*S*°/*R* + ln*Φ*. Since the value of the phase ratio is seldom known, Δ*S*°* values (Δ*S*°* = Δ*S*° + *R*ln*Φ*) are often used to compensate for the uncertainty in *Φ* [38,39].

Similarly, the differences in the change of standard enthalpy Δ(Δ*H°*) and standard entropy Δ(Δ*S°*) for the two enantiomers moving from the mobile phase to the stationary phase were also calculated according to the modified van ’t Hoff equation:

(2) $$\ln\alpha =-\frac{∆\left(∆H^{{}^{\circ}}\right)}{RT}+\frac{∆\left(∆S^{{}^{\circ}}\right)}{R}$$

The isoenantioselective temperatures (*T_iso_*) were also calculated as the ratio between Δ(Δ*H°*) and Δ(Δ*S°*), according to Equation (3)*. T_iso_* represents the temperature at which the enthalpy contribution is compensated by the entropic term, which means that Gibbs’ enthalpy (Δ(Δ*G*°)) equals zero. The thermodynamic parameter Δ(Δ*G*°) provides information on the strength of binding between selector and selectant, with more negative values indicating a more efficient binding. Consequently, a value of Δ(Δ*G*°) = 0 means that there is no difference between the binding strength of the enantiomers; therefore, at *T_iso,_* the two enantiomers co-elute, and no separation is achieved.

(3) $$T_{iso}=\frac{∆\left(∆H^{{}^{\circ}}\right)}{∆\left(∆S^{{}^{\circ}}\right)}$$

(4) $$∆\left(∆G^{{}^{\circ}}\right)=∆\left(∆H^{{}^{\circ}}\right)-T*∆\left(∆S^{{}^{\circ}}\right)$$

The obtained thermodynamic data are summarized in Table 2.

The results of the thermodynamic analysis revealed that the retention factors decreased as the temperature increased, and the selectivity values followed the same trend. The linear relationships indicated that the retention of the enantiomers occurred via a single associative mechanism and that the solvation–desolvation equilibrium did not obscure the association process of the analytes with the stationary phase [3,40]. The *Q* value, which is used for visualizing the relative contribution of enthalpic and entropic terms to the free energy of adsorption, was higher than 1, indicating that enthalpic control was mainly responsible for the separation of the APR enantiomers, which is usually the case for protein-based columns. The *T_iso_* value was too high to be of practical importance; however, theoretically, further raising the temperature above the *T_iso_* value would reverse the elution order, due to the greater entropic contribution. The high difference between the standard enthalpies (Δ(Δ*H*°) > 1 kJ/mol) showed a higher affinity and preferential binding of the *S*-enantiomer to the chiral selector, when compared to its antipode.

#### 2.1.4. Characterization of the APR-HSA Binding by HPLC

Chromatographic columns with HSA can be used to determine the binding percentage of different drugs. Different organic mobile phase modifiers were used and compared during this study for the analysis of their effect on the calculated bound% [16,30].

As it was observed, the decrease of the organic modifier percentage in the mobile phase caused an exponential increase in the retention times of both enantiomers. Therefore, for the calculations, the logarithm of the retention factors was plotted against the organic modifier proportion. With this conversion, a linear relationship was obtained, which could be easily extrapolated to zero for supposedly the best imitation of in vivo conditions. As the intercept of these linear equations, a *k* value is expressed that can be used to calculate the bound% according to Equation (7).

Using this method, the calculated bound% data are shown in Table 3.

The data showed that APR binding to HSA was significant, as the bound% value was higher than 90% for both enantiomers. From the obtained data, it can also be observed that the obtained values were similar, indicating that the organic modifier did not significantly influence the binding study of APR to HSA.

### 2.2. Fluorescence-Based Binding

**Figure 4:**
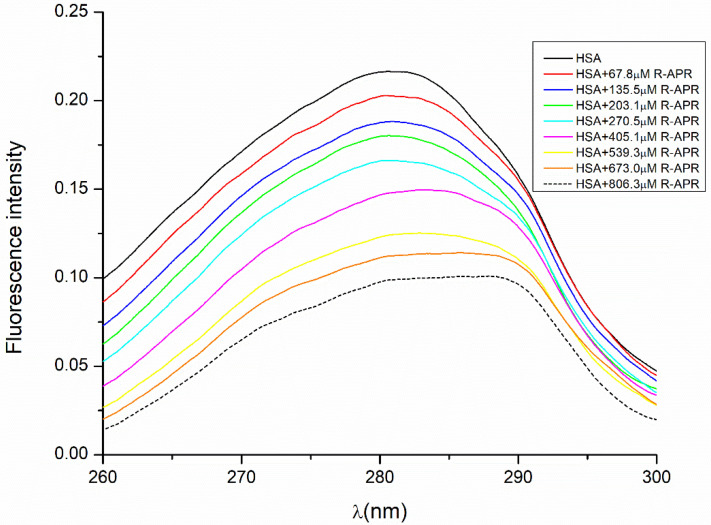
Quenching of HSA fluorescence by *R*-APR.

Fluorescence quenching is a popular method for the study of molecular interactions between proteins and drugs because it is sensitive, rapid and easy to use [41,42,43,44]. In this study, the fluorescence spectroscopic method was used to determine the stability constants of both enantiomers complexed with albumin to interpret the enantiospecific difference of HSA binding. The emitted fluorescence of HSA is primarily caused by amino acids with aromatic side chains, their specific location within the protein and their interactions. When the native structure is disrupted by a drug interacting with the protein, usually a decrease in the native fluorescence can be observed. Therefore, as expected when HSA was titrated with increasing concentrations of APR, a quenching could be observed in the fluorescence of HSA. The molecular mechanisms that lead to fluorescent quenching are excited state reactions, molecular rearrangements, transfer of energy and static and dynamic quenching [45]. To avoid any error, the fluorescence of the actual concentration of APR was measured as well and subtracted from the fluorescence of the complex. The resulting spectra in the case of *R*-APR are shown in Figure 4, where the cumulative emitted fluorescence intensity is plotted against the excitation wavelength. The maximum fluorescence intensity of native HSA was determined at an excitation wavelength of 280 nm; therefore, the quenching data were measured at this wavelength. While increasing the APR concentration, a progressive decrease was observed in the fluorescence intensity of HSA. Because the structure of HSA presents a single Trp (Trp-214), the decrease could be due to the altered microenvironment of the Trp-214.

The observed fluorescence intensities of HSA and the HSA–APR complexes at increasing APR concentrations were plotted against the total concentration of APR in the solution, and the stability constants were calculated according to Equation (18) by nonlinear parameter fitting. The equation does not contain any simplifications; it can be used universally.

The calculated stability constants (*K_R_*_-APR-HSA_ = 6.45 × 10^3^ M^−1^ and *K_S_*_-APR-HSA_= 1.04 × 10^4^ M^−1^) indicated that the strong interaction between HSA and APR was responsible for the fluorescence quenching. The fluorescent data support the results acquired from the HPLC measurements and confirm that the chromatographic enantioseparation took place due to the different binding affinities of the enantiomers with HSA.

### 2.3. Molecular Docking Results

**Figure 5:**
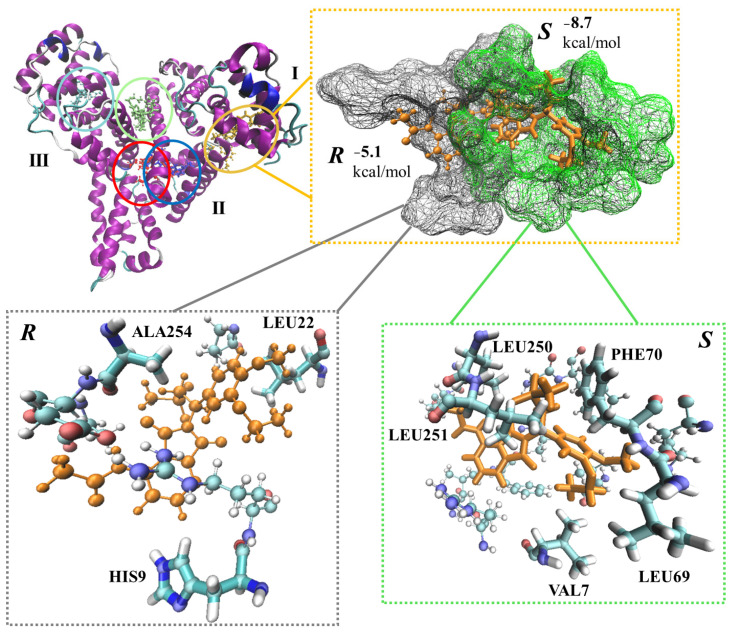
APR (*S*) and its enantiomer (*R*) docked to potential binding sites of human serum albumin (HSA, top left corner). I, II and III are HSA domains. The structures are highlighted in red, blue, lime, yellow and cyan. The most favored APR binding was selected along with the corresponding enantiomer (top right corner). Amino acid residues within 2 Å of the ligands are highlighted (bottom).

All in all, five binding sites were identified in HSA and *S*-APR, and its isomer were docked into each of those (Figure 5).

The least and most favored binding of APR on HSA deviated by almost 5 kcal/mol in terms of docking scores (Table 4, Figure 5, lime and yellow, respectively).

Two of the identified potential binding sites somewhat overlapped, and thus, the docking to those resulted in overlapping APR structures (Figure 5, red and blue ligands). These differed in terms of the corresponding docking scores by >2.5 kcal/mol (Table 4, *S*-APR). The most preferred binding (yellow) of the eutomer (*S*-enantiomer) was lower by 1.68 kcal/mol compared to the most favored binding of the distomer (*R*-enantiomer) (red) in terms of docking score (Table 4), which is in excellent agreement with the experimental findings. The position of the *S*-enantiomer with the most favored docking score (yellow) was further analyzed and compared with the position of the *R*-enantiomer (Figure 5, top right corner). HSA is divided into three main domains, and the most favored APR binding occurred at the border between domains I and II (Figure 5, top right corner). The binding pocket around the *S*-enantiomer is more compact, with five amino acid residues within 2 Å (VAL7, LEU69, PHE70, LEU250 and LEU251) compared to the binding site for the *R*-enantiomer, which only includes three residues (HIS9, LEU22 and ALA254) within this distance (Figure 5, bottom). Thus, more interactions occur between HSA and the *S*-enantiomer, including a T-shaped π-stacking, which involves PHE70 and the phenyl group of the drug (Figure 5, bottom right corner). The occurrence of a π–π interaction is well in line with the predicted atomistic details.

## 3. Materials and Methods

### 3.1. Materials

*R*- and *S*-APR were purchased from Beijing Mesochem Technology Co. Ltd. (Beijing, China). Human serum albumin was ordered from Tokyo Chemical Industry Co. Ltd. (Tokyo, Japan). Methanol (MeOH), ethanol (EtOH), 1-propanol (PROP), 2-propanol (IPA) and acetonitrile (ACN) of gradient grade were purchased from Merck (Darmstadt, Germany)**.** The immobilized Chiral-HSA column (4.0 × 100 mm, 5 μm) was ordered from Chiral Technologies Inc., a subsidiary of Daicel Chemical Industries Ltd. (Tokyo, Japan). Analytical-grade acetic acid, phosphate salts, phosphoric acid, sodium hydroxide and ammonium–acetate were ordered from Sigma-Aldrich, Hungary (Budapest, Hungary). These were used for the preparation of the buffer solution. All reagents were used without further purification. The deionized water was prepared by a Milli-Q Direct 8 Millipore system (Millipore Corporation, Bedford, MA, USA).

### 3.2. HPLC

LC–UV analysis was carried out on an Agilent 1100 HPLC system consisting of a quaternary pump, an autosampler, a column compartment, and a DAD detector (Agilent Technologies, Waldbronn, Germany). ChemStation software (ver.: B04 03-SP2) was used for data analysis. UV detection was performed at 230 nm. The APR stock solution was prepared at 1 mg mL^−1^ in MeOH, and further dilutions were made with water. An injection volume of 5 μL was used.

The retention factor (*k*) was calculated using the following equation:

(5) $$k=\frac{t_{r}-t_{0}}{t_{o}}$$

where *t_r_* is the retention time of the analyte, and *t*_0_ is the dead time, determined as the first peak appearance in the chromatogram.

The separation factor (*α*) was calculated as follows:

(6) $$\alpha =\frac{k_{2}}{k_{1}}$$

where *k*_1_ and *k*_2_ are the retention factors of the first- and second-eluting enantiomer, respectively. The *k* values were used to calculate the albumin-bound percentage (*b%*) using Equation (7) by zonal elution chromatography [12]:

(7) $$b\%=\frac{k}{1+k}\cdot 100$$

### 3.3. Fluorescence Measurements

The fluorescence measurements were carried out on a Jasco J-815 spectropolarimeter with a connected CDF-426L fluorescence detector and a temperature-controlled 10 mm cuvette holder (Jasco, Tokyo, Japan), thermostated to 25 °C with a Lauda water bath circulation thermostat (Lauda Brinkmann, Königshofen, Germany). Spectra Manager software (ver.: 2.08.04) was used for data analysis. The fluorescence analysis was performed with an excitation spectrum of 260–300 nm and detection of the cumulative fluorescence spectrum. The HSA solution was prepared at 0.064 mg mL^−1^ in 10 mM phosphate buffer adjusted to pH 7. A volume of 3 mL of the HSA stock solution with an HSA concentration of 0.32 μM was titrated with 2.5–30 μL of *S*- and, separately, *R*-APR solutions prepared at 3.75 mg mL^−1^ in MeOH, which equals to a range of 6.8–80.6 μM of APR in the measured solution.

The stability constants of both enantiomers were calculated. The measured fluorescence signal (*I_obs_*_._) is the weighted average of the free protein fluorescence (*I_HSA_*) and the bound, complexed species fluorescence (*I_APR-HSA_*), as Equation (8) shows. Note that since the fluorescence signal of the ligand (*APR*) was subtracted for each sample, (*I_APR_*) could be excluded from this equation

(8) $$I_{obs.}=I_{HSA}\cdot \chi_{HSA}+I_{APR-HSA}\cdot \chi_{APR-HSA}$$

where *Χ_HSA_* is the mole fraction of free HSA, while *Χ_APR-HSA_* is the mole fraction of the APR-HSA complex. The molar fractions can be expressed as follows:

(9) $$\chi_{HSA}=\frac{\left[HSA\right]}{{\left[HSA\right]}_{T}}=\frac{{\left[HSA\right]}_{T}-\left[APR-HSA\right]}{{\left[HSA\right]}_{T}}$$

(10) $$\chi_{APR-HSA}=\frac{\left[APR-HSA\right]}{{\left[HSA\right]}_{T}}$$

The square brackets denote the equilibrium concentration of the species in molarity, while the ‘*T*’ subscript represents the total (analytical) concentration of *APR* or *HSA* present, i.e.:

(11) $${\left[HSA\right]}_{T}=\left[HSA\right]+\left[APR-HSA\right]$$

(12) $${\left[APR\right]}_{T}=\left[APR\right]+\left[APR-HSA\right]$$

The law of mass action for the protein binding can be written as follows:

(13) $$K=\frac{\left[APR-HSA\right]}{\left[APR\right]\cdot \left[HSA\right]}$$

which in turn can be combined with Equations (11) and (12) to obtain the following expression:

(14) $$K\cdot \left({\left[APR\right]}_{T}-\left[APR-HSA\right]\right)\cdot \left({\left[HSA\right]}_{T}-\left[APR-HSA\right]\right)=\left[APR-HSA\right]$$

Rearranging expression (10) leads to a quadratic expression for [APR-HSA]:

(15) $${\left[APR-HSA\right]}^{2}+\left(-{\left[APR\right]}_{T}-{\left[HSA\right]}_{T}-\frac{1}{K}\right)\cdot \left[APR-HSA\right]+{\left[APR\right]}_{T}\cdot {\left[HSA\right]}_{T}=0$$

The solution to the above quadratic equation gives rise to only one real solution of chemical relevance:

(16) $$\left[APR-HSA\right]=\frac{\left({\left[APR\right]}_{T}+{\left[HSA\right]}_{T}+\frac{1}{K}\right)-\sqrt{{\left({\left[APR\right]}_{T}+{\left[HSA\right]}_{T}+\frac{1}{K}\right)}^{2}-4\cdot {\left[APR\right]}_{T}\cdot {\left[HSA\right]}_{T}}}{2}$$

Finally, by combining Equations (8)–(10), we can express the observed fluorescence intensity as follows:

(17) $$I_{obs.}=I_{HSA}\cdot \frac{{\left[HSA\right]}_{T}-\left[APR-HSA\right]}{{\left[HSA\right]}_{T}}+I_{APR-HSA}\cdot \frac{\left[APR-HSA\right]}{{\left[HSA\right]}_{T}}=I_{HSA}-\left(I_{HSA}-I_{APR-HSA}\right)\cdot \frac{\left[APR-HSA\right]}{{\left[HSA\right]}_{T}}$$

and inputting the expression of Equation (16) into Equation (17) provides us with an expression for *I_obs_*_._ in terms of parameters that can be fitted (analytical concentrations of *APR* and *HSA*, stability constant, and limiting fluorescence intensity of *HSA* and the complex) using regression analysis:

(18) $$I_{obs.}=I_{HSA}-\left(I_{HSA}-I_{APR-HSA}\right)\cdot \frac{\left({\left[APR\right]}_{T}+{\left[HSA\right]}_{T}+\frac{1}{K}\right)-\sqrt{{\left({\left[APR\right]}_{T}+{\left[HSA\right]}_{T}+\frac{1}{K}\right)}^{2}-4\cdot {\left[APR\right]}_{T}\cdot {\left[HSA\right]}_{T}}}{2\cdot {\left[HSA\right]}_{T}}$$

The stability constants were calculated by non-linear parameter fitting by Origin software (OriginPro 8.5, OriginLab Corporation, Northampton, MA, USA).

### 3.4. Docking Study

The calculations were carried out using the Schrödinger suite (Release 2020-4). Due to the fact that the APR-HSA complex is not available in the Protein Data Bank (PDB), an HSA structure—PDB ID: 1H9Z [46]—with appropriate resolution (2.5 Å) was selected, which was applied in previous studies [47,48,49]. The 3D structure was imported into the Protein Preparation Wizard [50], non-protein species were deleted, and the protein was prepared by using default settings. The preparation process included the addition of hydrogen atoms to the HSA model, after which PROPKA was employed to predict the protonation state of the residues at physiological pH [51], and the system was minimized by using the OPLS3e force field [52]. Potential binding sites on HSA were identified using SiteMap [53,54]. The ligand structure was prepared using the LigPrep module, while the Receptor Grid Generation module was used to specify the target area by applying the SiteMap results. Flexible molecular docking was performed using the extra precision (XP) mode of Glide [55,56,57]. Visualization of the structures was carried out by using VMD 1.9.3 [58].

## 4. Conclusions

In the present work, the enantioselective, in vitro HSA binding of APR was investigated. Using state-of-art techniques, the APR-HSA complex was characterized at the molecular level. The development of fast enantioselective methods for the characterization of protein binding is crucial in the early stage of drug development, because the different HSA binding of enantiomers is necessarily associated with different pharmacokinetic properties. We found that the eutomer *S*-APR bound stronger to HSA than its antipode, mainly due to an extra secondary interaction between *S*-APR and PHE70 in HSA. It can also be seen that complementary techniques such as chromatography, spectroscopy and computational methods should be applied for an accurate and detailed characterization. Chromatographic methods can be used to quantify the enantioselective binding and to foretell in vivo the binding to HSA of the individual enantiomers, while fluorescence spectroscopy can be used to determine the stability constants of the enantiomer–HSA complexes. Docking methods can reveal the binding regions and secondary interactions between the carrier protein and individual enantiomers. However, it should be noted that in vitro and in vivo data may differ. The equilibrium and structural information presented in our work also offer a molecular basis for a better understanding of APR pharmacokinetic properties. Our general method could be applied for other chiral drugs, for which the enantiospecific pharmacokinetic parameters could be important.

## Figures and Tables

**Table 1:** Retention factors, resolutions and selectivity values in different percentages of five different organic modifiers on the Chiral-HSA column.

Organic Modifier	%	*k_R_*_-APR_	*k_S_*_-APR_	*R*_s_	*α*
MeOH	2	13.05	19.00	1.78	1.46
	4	9.32	15.27	1.56	1.63
	6	6.05	8.07	1.01	1.34
	8	4.48	5.32	0.65	1.19
	10	3.72	3.98	0.32	1.07
EtOH	2	8.72	13.84	1.93	1.59
	4	4.93	6.52	1.32	1.32
	6	3.33	4.12	0.85	1.24
	8	2.83	3.20	0.60	1.13
	10	2.66	2.94	0.32	1.10
1-PROP	2	8.99	13.98	1.36	1.55
	4	5.96	8.04	0.99	1.35
	6	4.19	5.20	0.62	1.24
	8	1.99	2.25	0.31	1.13
	10	1.32	1.32	0	1
IPA	2	8.04	13.48	1.94	1.68
	4	5.00	7.62	1.43	1.52
	6	3.01	3.76	1.19	1.24
	8	2.26	2.56	0.65	1.13
	10	1.45	1.45	0	1
ACN	2	7.56	10.05	1.46	1.33
	4	3.98	4.90	0.69	1.23
	6	2.80	3.09	0.31	1.11
	8	2.03	2.03	0	1
	10	1.42	1.42	0	1

**Table 2:** Calculated thermodynamic parameters of APR enantiomers on the Chiral-HSA column in 10 mM phosphate buffer/IPA 96/4 *v/v*% mixture.

Enantiomer	Equation	r^2^	Δ(Δ)*H*° (kJ/mol)	Δ(Δ)*S*° (J/molK)	Δ(Δ)*G*° (kJ/mol)	*T_iso_* (°C)	*Q* *
*R*-APR	ln*k*_1_ = 3189.8x − 9.176	0.9994	−26.52	−76.29	−3.79		
*S*-APR	ln*k*_2_ = 3641.8x − 10.438	0.9997	−30.28	−86.78	−4.42		
	ln*α* = 452.01x − 1.2619	0.9881	−3.76	−10.49	−0.63	85.20	1.2

* $Q=\frac{\Delta \left(\Delta H^{{}^{\circ}}\right)}{T\cdot \Delta \left(\Delta S^{{}^{\circ}}\right)}$ at 298 K.

**Table 3:** Bound percentage values (*b*%) determined for the extrapolation to zero percentages of different organic modifiers.

Organic Modifier	*b*%*_R_*_-APR_	*b*%*_S_*_-APR_
MeOH	94.5	96.8
EtOH	91.4	94.8
1-propanol	94.0	96.4
IPA	92.1	95.7
ACN	91.3	93.7
Average + SD%	92.7 ± 1.5	95.5 ± 1.3

**Table 4:** Docking scores of APR at potential human serum albumin (HSA) binding sites.

Docking Score of *S*-APR (kcal/mol)	Docking Score of *R*-APR (kcal/mol)	Structures at the Potential Binding Sites *
−4.2	−7.1	red
−6.7	−6.8	blue
−3.8	−4.3	lime
−8.7	−5.1	yellow
−7.9	−3.2	cyan

* The colors are assigned according to Figure 5.

## Data Availability

Not applicable.
